# Real-world evaluation of online visual acuity self-testing and remote consultation: three years of implementation experience in a paediatric ophthalmology service

**DOI:** 10.1038/s41433-026-04575-1

**Published:** 2026-06-03

**Authors:** Fengyi Liu, Raisa Ahmed, Bethany Ellis, Deborah Mullinger, Louise Allen

**Affiliations:** 1 School of Clinical Medicine, University of Cambridge, Cambridge, UK; 2 Department of Orthoptics, Cambridge University Hospitals NHS Foundation Trust, Cambridge, UK; 3 Department of Ophthalmology, Cambridge University Hospitals NHS Foundation Trust, Cambridge, UK

**Keywords:** Health services, Diagnosis

## Abstract

**Background/Objectives:**

Our previous validation studies indicated that automated best corrected visual acuity (BCVA) testing using the DigiVis web application is comparable to standard testing. Here, we evaluate real-world experience of digital BCVA self-testing (DVA) and remote consultation (RC) in a paediatric ophthalmology service.

**Methods:**

Electronic records of children assigned follow up RC with home DVA testing for their routine clinical care between March 2021 and October 2024 were reviewed. Demographic and clinical features, test duration and usability scores were analysed. Serial BCVA and DVA results were compared using Bland Altman statistical methods. Clinical outcomes and service adaptations were assessed.

**Results:**

205 RC appointments with DVA testing were scheduled for children (aged 4–14, median 6 years). DVA results were available for 192 (93.7%) RCs, with 166 (86.5%) tests undertaken without clinical supervision. The mean bias between serial BCVA and DVA was 0.005 logMAR (*p* < 0.001) with upper and lower limits of agreement of +0.182 (95%CI: 0.169 to 0.195) and –0.173 (–0.186 to –0.160) logMAR respectively. Expedited follow-up face-to-face consultations (f2fC) were arranged for 13 families unwilling or unable to self-test, 5 children with poor concentration and 9 with self-detected deterioration of BCVA. Informative DVA results were available for 187 (91.2%) encounters. 116 (81.7%) of 142 families voluntarily completing Likert scoring rated the application good/excellent.

**Conclusion:**

In this real-world evaluation, 91.2% of offered DVA tests were informative and contributed to clinical decision making. DVA correctly identified unexpected deterioration in 9 children supporting the utility of this innovative service model.

## Introduction

The Covid pandemic disrupted working patterns and particularly affected ophthalmology, the service with the largest hospital out-patient workload (approximately 9 million appointments per year) [1]. Long clinic backlogs currently cause a delay in diagnosis and are responsible for avoidable vision loss in hundreds of patients annually [2]. With a 40% increase in demand expected over the next 20 years, innovative strategies are needed to increase the capacity within hospital eye services [3, 4].

Remote (video or telephone) consultation (RC) can improve the efficient use of staff and clinical space and are generally popular with patients [5]. Advantages include fewer hours off work or school, less travel and better access to services for those in rural locations. Potential disadvantages include a perception of transactional or depersonalised care and a reduction in the therapeutic relationship between clinician and patient or parent. Currently, only 5% of eye consultations are undertaken remotely compared to the NHS target of a third. The current inability to conduct a formal vision measurement to inform the remote consultation is a limiting factor [6].

Best corrected distance visual acuity (BCVA) monitoring is fundamental for children at risk of, or being treated for, amblyopia. Frequent clinic attendances, primarily to assess changes in BCVA, are needed and can be onerous for families. There is the potential to replace a proportion of these face-to-face consultations (f2fC) with RC and self-tested BCVA at home.

For this solution to be effective, accurate home BCVA testing is needed. Although hundreds of vision testing applications (apps) are available, few are clinically validated or registered for medical use, and most require the input of a trained observer. Previous small-scale studies have reported varying reliability of home vision testing using a handful of commercially available apps, none of which have been adopted into regular service workflows [7, 8].

DigiVis DVA is a web app enabling automated digital self-testing of BCVA (DVA), which our previous validation studies have reported to have similar accuracy to standard testing in this age group [9, 10]. The prototype app was introduced in our unit for RC during the Covid pandemic in 2021. The initial concept has been further developed, iteratively improved with clinical use and feedback and integrated into the orthoptic and paediatric ophthalmology clinical pathways in our NHS trust. We report our experience with DVA and RC in our paediatric ophthalmology service over a 43-month period.

## Materials and methods

### Participants

This was an observational longitudinal review of the electronic patient records (EPR) and DigiVis portal metadata of children who had DVA and RC as part of their standard clinical management plan between March 2021 and the end of September 2024. Suitability for video or telephone review was a pragmatic one, made by the clinician at the time of the child’s comprehensive f2fC. The agreed departmental guidelines for suitability included: children aged between 4 and 16 likely to be able to comply with testing, BCVA in the worse eye better than +0.80 logMAR, no requirement for a repeat full orthoptic assessment, conversational English ability, digital access at home for DigiVis DVA (WIFI and two smart devices) and parental agreement. Parents were given an NHS trust-approved clinical patient information leaflet, an occlusive patch to aid home testing and a paper tape measure if required. Parents were either given a password (from 2021 to February 2023) or sent a link by SMS and email to access the online vision test (from March 2023 onwards). All families scheduled for DVA and RC over the 43 month evaluation period were included in the data analysis.

As a service evaluation of fully established treatment pathway in our NHS trust, study approval was provided by the trust’s Quality and Safety Committee and regional ethics committee approval was not required.

### The technology

**Fig. 1:**
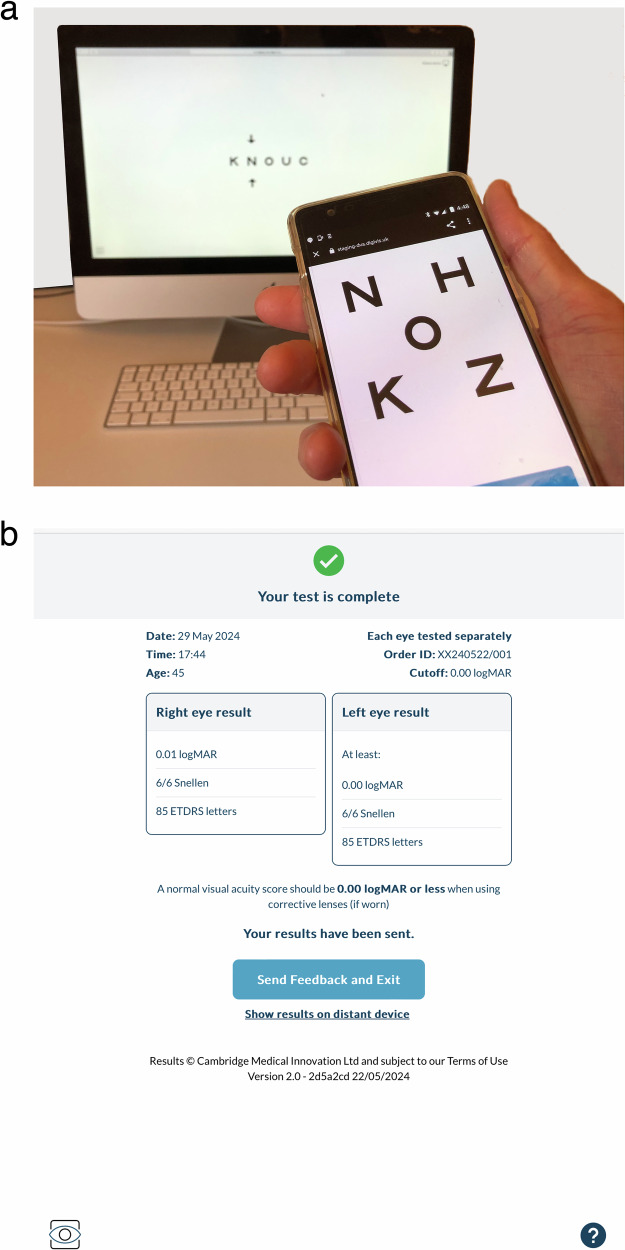
DigiVis DVA in use. **a**, **b** The patient is positioned 2 m away from the distant device. **a** The linear Sloan letter display with crowding arrows indicating the letter the patient should match. **b** The result screen on the patient’s hand-held device.

DigiVis DVA runs on two digital devices (e.g. smartphones) paired over the internet. No download is necessary, either on the patient’s devices or the hospital IT systems. The test mimics a matching linear logMAR flipchart, with the child selecting the one letter out of the 5 displayed on their handheld device which matches the Sloan optotype letter indicated in the logMAR row on the second device, 2 metres away (Fig. 1a). An embedded instruction video on the landing page of the app demonstrates the steps for screen calibration, measuring the viewing distance, pairing the devices and undertaking the test itself. Where fewer than 5 letters can be displayed on the patient’s distant display screen (for instance, for larger letter sizes or when a mobile phone is used), a crowding box surrounds the letter. As a simplification for 4-year-olds, a single letter (HTOV or X) in a crowding box is used instead of the linear letter display. The app is gamified for children with cartoon animals collected after each response. A proprietary staircasing algorithm is used to calculate VA threshold [11]. Although the test VA threshold is configurable for each test down to a threshold of -0.30 logMAR, a cut-off of 0.00 logMAR was the default setting used in this paediatric population, to minimise test duration. Where no incorrect matches (reversals) are made, 5 letters must be matched correctly at the chosen cut-off level. The parent and child decide which eye to test first.

Results are calculated in logMAR units but additionally converted and displayed in Snellen and ETDRS (Early Treatment of Diabetic Retinopathy Study) notations (Fig. 1b) on the handheld device. The parent keeps their child’s results, which can be recorded with a screenshot or sent to their phone by SMS or email. Following completion of testing, the user is invited to rate the acceptability of the app using a visual Likert scale and to leave feedback. The clinician can independently look up the test result on the portal using the test order number.

DigiVis DVA is registered as a Class 1 medical device under UK MDR (UKCA marked) and is compliant with the NHS DCB0129 and DCB0160 clinical risk management standards.

### Analysis

To account for expected improvement in serial BCVA assessment with amblyopia therapy, the DVA was compared to the mean of the BCVA assessments at the preceding and subsequent clinic visits where applicable. Where f2f BCVA was recorded as <0.00 logMAR, the value was rounded up to 0.00 to enable comparability with DVA scores. Using a linear mixed-effects model to account for eye co-dependence and individual differences in ability, agreement between the measurements was evaluated using Bland-Altman plots, looking specifically at mean bias and 95% limits of agreement (LOA). Intraclass correlation coefficients (ICC) was also calculated. A sub-group analysis compared agreement in amblyopic eyes versus partner non-amblyopic eyes in children having treatment for amblyopia using Levene’s test and paired *t*-test calculations. Assumptions for the paired *t*-test were checked and met. A priori standards were used to facilitate appraisal of agreement as quantified by ICCs. Children’s age at the time of their DVA, their test duration and voluntary visual Likert acceptability scores (retrieved from app metadata) were calculated using descriptive statistics.

## Results

### Patient demographics

A total of 205 RC with DVA encounters took place over the 43-month review period, with 4 children having two recorded encounters. The age range was 4–14 years (mean 6.32 years, median 6 years). 75 (36.6%) of the encounters took place during active occlusion therapy for amblyopia. The most common primary diagnoses were strabismus in 73 (35%), anisometropia in 66 (32%) and other refractive errors in 30 (14%).

### Remote consultation outcome

**Fig. 2:**
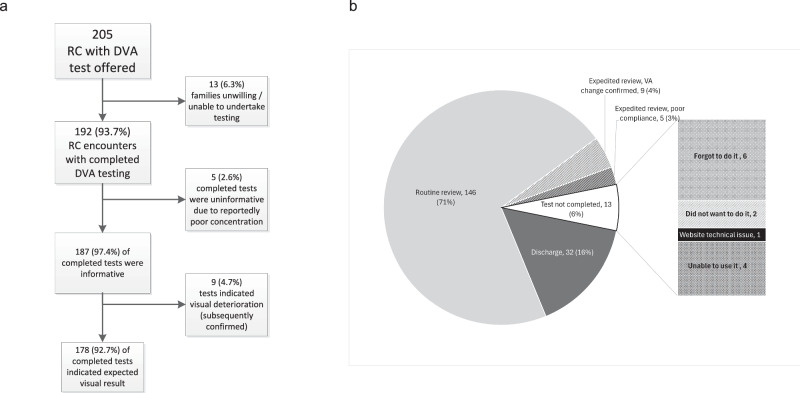
Remote consultation outcome. **a** Encounter flow diagram of remote consultations (RC) and self tested digital best corrected visual acuity assessments (DVA). **b** Outcome of the remote consultation (*n* = 205).

DVA testing was completed for 192 (93.7%) RC encounters but was unreliable due to reported or witnessed poor concentration in 5 of these, leaving 187 (91.2%) of the 205 RC appointments with an informative DVA assessment available for clinical decision (Fig. 2a). The clinical outcome of the RC is illustrated in Fig. 2b. In total, expedited f2fC were given after 27 (13.2%) RCs: in 13 cases because the test was not undertaken, in 5 children with poor concentration (all 5- or 6-year-olds), and in a further 9 cases for an unexpected deterioration apparent in their DVA (subsequently confirmed at f2fC). A decision to discharge the child to community care was made after 32 (15.6%) RC and the remainder were given routine reviews (90.4% of which were f2fC).

### Test duration and patient acceptability

**Fig. 3:**
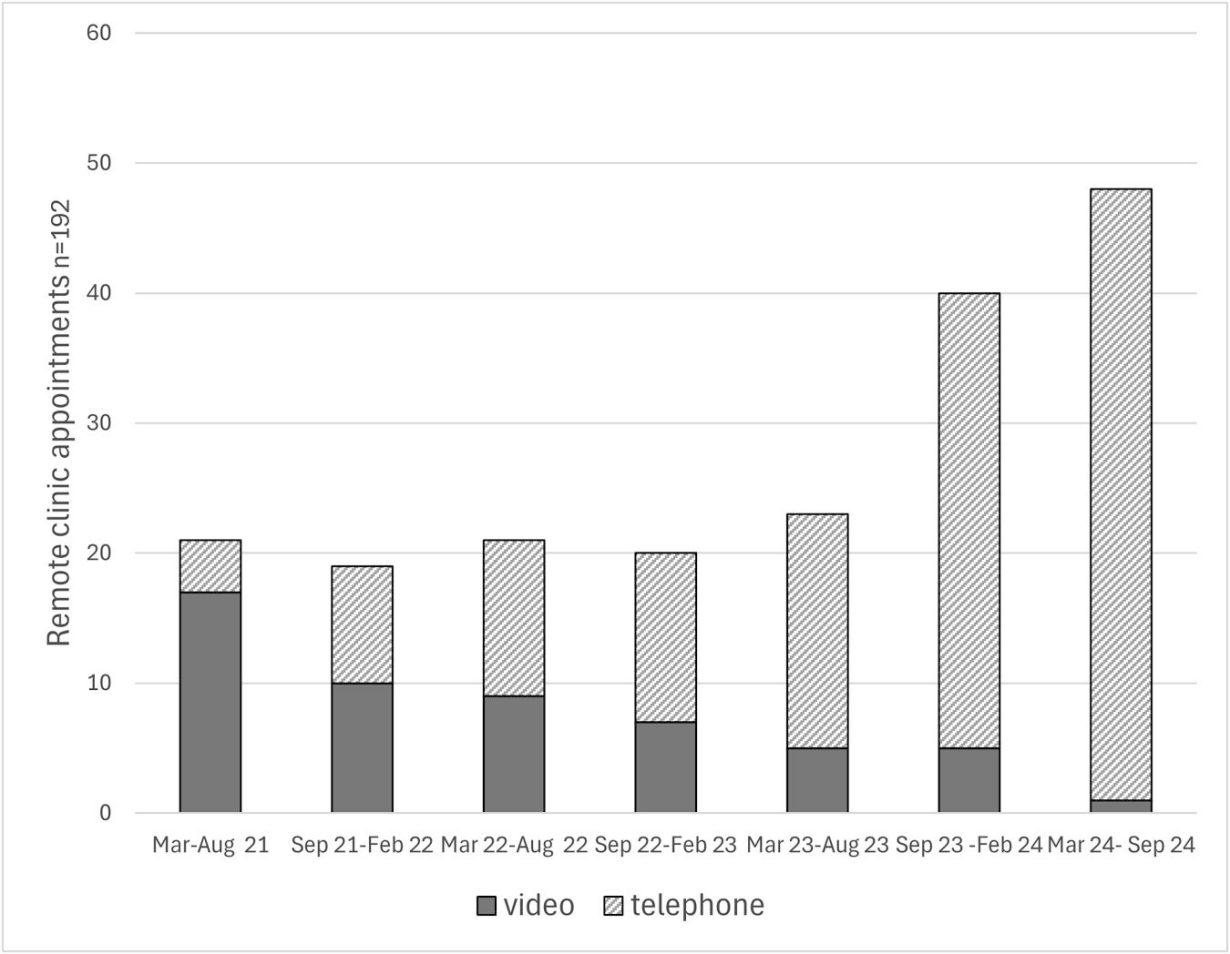
Number of telephone and video remote consultations. Histogram illustrating relative numbers of video and telephone over the implementation period.

Mean DVA test duration (including any requested test pauses) was 7.8 minutes ±SD 3.9 minutes (range 2.06–14.18 mins, median 7.5 minutes). 142 (74%) of the 192 DVA tests had completed voluntary visual Likert scores, with 116 (81.7%) ratings equivalent to good or excellent. Documented in the electronic records were: 8 comments suggesting that the children really enjoyed/were keen to do the test /preferred the test to standard testing, 21 indicating that the test was easy to set up and 4 comments reporting difficulty setting up the test. The proportion of RC undertaken by telephone rather than video increased from 14.3% to 97.9% over the 43-month study period (Fig. 3).

### Serial clinic and DVA test results

F2f and DVA results for 383 eyes were available for analysis; one amblyopic eye was excluded from analysis because the initial f2f BCVA was worse than +0.8 logMAR. The range of initial BCVA was 0.00 to +0.675 logMAR (interquartile range + 0.138 logMAR, mean 0.092 ± 0.128 logMAR). 26 (13.5%) of DVA tests were undertaken under video supervision using the shared screen function, the remainder were undertaken asynchronously, before the telephone consultation.

**Fig. 4:**
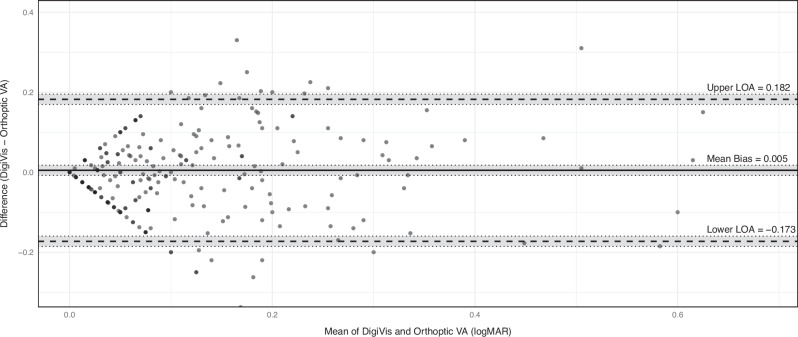
Serial clinic and DVA test results. Bland Altman plot comparing self-tested digital best corrected visual acuity (DVA) serial face-to-face best corrected visual acuity (BCVA). The bias is indicated by solid line and 95% LOA is indicated by dashed line, whilst shaded 95% confidence intervals for each are bounded by dotted lines.

Bland-Altman analysis compares the difference between DVA and clinic BCVA assessments to their mean. Points plotted above and below 0.00 logMAR indicate an under-estimation and over-estimation by DVA testing respectively (Fig. 4). For the population studied, the mean bias was not statistically significantly different from 0.00 logMAR as indicated by the inclusion of x-axis within confidence intervals. The combined upper and lower 95% limits of agreement (LOA) between f2f and DVA assessments were +0.182 and –0.173 logMAR (Table 1). Good agreement between f2f and DVA is indicated by ICC values of 0.745 (*p* < 0.001). Using a linear mixed-effect model to account eye co-dependence and individual variability in test performance, there was no evidence for a significant difference between testing method (estimated effect = 0.0048, *p* = 0.467) or tested eye (estimated effect = –0.0034, *p* = 0.601).

**Table 1:** Bland Altman calculations of mean bias, upper and lower 95% Limits of Agreements (LOA) comparing self-tested digital best corrected visual acuity (DVA) serial face-to-face best corrected visual acuity (BCVA) with Intraclass Correlation Coefficients (ICC).

	Number(eyes)	Mean bias (95% CI) logMAR	Upper LOA(95% CI) logMAR	Lower LOA(95% CI) logMAR	ICC(95% CI)	*P* value
Right eyes	192	–0.001 (–0.013 to 0.011)	+0.164 (0.152 to 0.176)	–0.167 (–0.179 to –0.155)	0.746 (0.676 to 0.803)	<0.001
Left eyes	191	–0.008 (–0.020 to 0.004)	+0.154 (0.142 to 0.165)	–0.170 (–0.182 to –0.158)	0.746 (0.676 to 0.803)	<0.001
Amblyopic eyes	74	–0.029 (–0.050 to –0.009)	+0.146 (0.126 to 0.167)	–0.205 (–0.226 to –0.185)	0.832 (0.734 to 0.894)	<0.001
Non-amblyopic partner eyes	75	–0.008 (–0.020 to 0.003)	+0.090 (0.078 to 0.101)	–0.106 (–0.117 to –0.095)	0.432 (0.231 to 0.598)	<0.001
All eyes	383	0.005 (–0.008 to 0.018)	+0.182 (0.169 to 0.195)	–0.173 (–0.186 to -0.160)	0.745 (0.697 to 0.787)	<0.001

Sub-group analysis demonstrated a higher absolute mean bias in the amblyopic eyes compared to non-amblyopic eyes (paired *t*-test, *t* = –2.15, *p* = 0.035). The LOA width was significantly more variable in amblyopic eyes (*F* = 26.27, *p* < 0.001) but the ICC of 0.832 (95%CI 0.734 to 0.894) indicated good agreement between the two methods of testing.

## Discussion

To our knowledge, this is the first real-world longitudinal evaluation of serial f2f and DVA assessments and RC used for ophthalmic out-patient service provision. The study indicates that informative and accurate DVA assessments were completed in 91.2% of 205 RC encounters where it was offered and supported clinical decision making. Good or excellent acceptability was reported by 81.7% of families giving feedback.

BCVA in individuals with stable vision is expected to change by less than two lines (0.20) logMAR on repeat clinical testing [12]. Conventional ETDRS chart assessment in children aged 6–11 years with BCVA of +0.20 logMAR or better reported test-retest LOA of ±0.15 logMAR [13]. These LOA provide a standard against which a significant change in BCVA can be identified and digital vision tests can be assessed. For this cohort of 192 children having serial assessments, DVA values were found to have an insignificant mean bias and LOA of ±0.18 logMAR compared to serial f2f BCVA assessments. This level of accuracy falls within the expected variability of ETDRS testing and is similar to that found in previous two validation studies of the application [9, 10]. DVA assessment in this study supported decision-making during RC in all but 8.7% of families who, due to technical or concentration difficulties, had no informative testing and required expedited f2fC. DVA testing correctly identified unexpected visual deterioration in 9 children.

Potential aspects of home vision testing may affect its reliability: the ability of an untrained observer assessing BCVA accurately and the potential inaccuracies of test set-up at home—including screen calibration, viewing distance measurement and eye occlusion. Studies indicate that parents and non-clinical observers may underestimate BCVA when using iSight Pro and Peek Acuity (LOA range ± 0.44 to ± 0.74 logMAR) [7, 14, 15]. Automation can reduce the effect of observer bias and improve accuracy, as demonstrated in validation studies of DigiVis DVA and M&S EyeSimplify (LOA range ± 0.16 to ± 0.20 logMAR) [9, 10, 16]. To mitigate concerns about test set-up, several apps utilise the shared screen function over video-link, a trained observer supervising calibration and determining the BCVA. Levels of accuracy using this technique range from LOA of ±0.12 logMAR (COMPLog) to ± 0.35 logMAR Optonet and IbisVision) [8, 17]. Clinician uptake of the DVA and RC service was cautious in these first 43 months of implementation and all families who agreed to use the service were included in the data analysis. The clinicians’ initial preference was for supervised testing over video-link, but there was a shift to 97.9% asynchronous DVA and telephone consultation by the end of the study period. This suggests a growing clinical confidence in the test itself and ability for parents to set-up the test accurately without support. Additional influences may have been the introduction of a patient-facing instruction video, the templated duration of initiating consultation (15 rather than 30 minutes) and parental preference for their child not to miss school and to have a simple telephone call at their workplace.

The mean duration of the test was 7.8 minutes, the longest test recorded as 14.18 minutes in a 7-year-old child. This may not be an accurate reflection of the testing time itself since it included requested pauses. Although the app was given a good or excellent rating in 81.7% of uses, the voluntary nature of the visual Likert score completion may have biased results. The population using the service were the children of young parents with digital knowledge, this is likely to have been a factor in its acceptance and completion rate. Further exploration of barriers and facilitators to the uptake of this technology by older and digitally excluded patient groups is necessary.

Real-world studies are important for assessing implementation of validated technology in routine clinical practice and patient encounters, enabling the inclusion of patients who might otherwise not participate in research and informing iterative improvement of the technology. A disadvantage is that a stringent research protocol is not followed, and this can lead to incomplete and potentially unreliable data. Limitations of this study relate to its real-world nature: the decision of the clinicians to invite the family to have an RC was pragmatic and based on their perception of the child’s ability to undertake the test, as was their choice of test charts at the f2fC. F2f and DVA testing were not contemporaneous, but 3-4 months apart, preventing direct comparison, furthermore, 36.6% of the children were undergoing refractive adaption or having patching, with improving logMAR scores between consultations. For this reason, the DVA result was compared to the mean of the f2f BCVA undertaken in the months before and after the RC. Although an imperfect comparison, since the rate of BCVA improvement over the review period may not have been linear, the similarity of the mean bias and LOA in this study to previous validation studies suggests this was a reasonable assumption. A default cut-off threshold of 0.00 logMAR was used during this implementation period, although this minimised test duration, it prevented comparisons of intra-ocular BCVA difference in this study.

This longitudinal evaluation of automated home vision testing and RC provides real-world evidence of its potential. Innovative workflows using digital solutions are encouraged by NHS policy to meet ever increasing capacity pressures, improve equality of access to health care for rural populations and encourage active patient participation in healthcare, but are extremely slow to be implemented and adopted in the NHS [18]. Digitally excluded families are at higher risk of healthcare issues, and despite inequity of access to technology, they may benefit indirectly from improved service efficiency and prioritisation of access to timely f2f care [19].

The identification of staff, patient and organisational barriers and enablers for the uptake of medical technology are important to optimise use and reap the potential service and patient benefits. The co-design of an implementation model for home vision testing and remote consultation, and its evaluation in terms of service efficiency and carbon emission savings, is the subject of the team’s ongoing NIHR i4i Challenge funded study [20].

## Conclusion

This real-world longitudinal study suggests that digital visual acuity (DVA) self-assessment is a viable tool for paediatric ophthalmic remote consultations (RC). DVA testing was completed accurately in 91.2% of offered cases, supporting clinical decision-making and correctly identifying unexpected visual deterioration in nine children. Results showed an insignificant mean bias and limits of agreement (LOA) of ±0.18 logMAR compared to serial f2f assessments—a level of accuracy consistent with traditional ETDRS chart variability. While initial concerns regarding home set-up led to supervised video-link testing, a shift toward asynchronous testing (97.9%) by the study’s end indicates growing clinical confidence in parental ability to conduct these tests independently. Despite limitations such as non-contemporaneous comparison of visual acuity, high family acceptability (81.7%) and improved service efficiency highlight the potential for home vision testing and remote consultation to alleviate NHS capacity pressures.

## Summary

### What was known before?

- The NHS Long Term / 10 Year Plan target aims for a third of consultations to be undertaken remotely to ease capacity pressure.
- Studies of home vision testing using digital applications have showed variable levels of accuracy.

### What does this study add?

- Unsupervised self-testing of BCVA using an automated online application (DigiVis DVA) shows acceptable accuracy in real-world use and supports decision making during remote consultation.
- Home vision testing was rated good or excellent by 81.7% of patients and parents using this paediatric ophthalmology service.

## Data Availability

The de-identified study raw datasets generated during and/or analysed during the current study are available from https://osf.io/kz9gt/overview?view_only=6c41bf21ac074e6f8239205dcf00edb8.
